# Modified Split-Scan Computed Tomography (CT) Diagnostics of Severely Injured Patients: First Results from a Level I Trauma Center Using a Dedicated Head-and-Neck CT-Angiogram for the Detection of Cervical Artery Dissections

**DOI:** 10.3390/jcm9082568

**Published:** 2020-08-08

**Authors:** Daniel Popp, Claudius Thiedemann, Wolf Bäumler, Antonio Ernstberger, Volker Alt, Andreas Schicho

**Affiliations:** 1 Department of Trauma Surgery, University Medical Centre Regensburg, D-93053 Regensburg, Germany; daniel.popp@ukr.de (D.P.); Claudius.Thiedemann@stud.uni-regensburg.de (C.T.); antonio.ernstberger@ukr.de (A.E.); volker.alt@ukr.de (V.A.); 2 Department of Radiology, University Medical Centre Regensburg, D-93053 Regensburg, Germany; wolf.baeumler@ukr.de; 3 Department of Trauma and Hand surgery, Hospital Osnabrück, D-49076 Osnabrück, Germany

**Keywords:** cervical artery dissection, vertebral artery, carotid artery, polytrauma, severely injured, computed tomography

## Abstract

Introduction: Traumatic cervical artery dissections are associated with high mortality and morbidity in severely injured patients. After finding even higher incidences than reported before, we decided to incorporate a dedicated head-and-neck computed tomography angiogram (CT-A) in our imaging routine for patients who have been obviously severely injured or, according to trauma mechanism, are suspected to be severely injured. Materials and Methods: A total of 134 consecutive trauma patients with an ISS ≥ 16 admitted to our level I trauma center during an 18 month period were included. All underwent standardized whole-body CT in a 256-detector row scanner with a dedicated head-and-neck CT-A realized as single-bolus split-scan routine. Incidence, mortality, patient and trauma characteristics, and concomitant injuries were recorded and analyzed in patients with carotid artery dissection (CAD) and vertebral artery dissection (VAD). Results: Of the 134 patients included, 7 patients had at least one cervical artery dissection (CeAD; 5.2%; 95% CI 1.5–9.0%). Six patients (85.7%) had carotid artery dissections, with one patient having a CAD of both sides and one patient having a CAD and contralateral VAD combined. Two patients (28.6%) showed a VAD. Overall mortality was 14.3%, neurologic morbidity was 28.6%. None of the patients showed any attributable neurologic symptoms on admission. The new scanning protocol led to further 5 patients with suspected CeAD during the study period, all ruled out by additional magnetic resonance imaging with angiogram (MRI/MR-A). Conclusion: A lack of specific neurologic symptoms on admission urges the need for a dedicated imaging pathway for severely injured patients, reliable for the detection of cervical artery dissections. Although our modified CT protocol with mandatory dedicated CT-A led to false positives requiring additional magnetic resonance imaging, it likely helped reduce possible therapeutic delays.

## 1. Introduction

Overlooked injuries in severely injured patients are feared complications in the acute care of patients. Euler et al. were able to show that in 25% of cases at least one diagnosis remains undiscovered in severely injured patients [1]. With a mortality rate of up to 33% and a neurological morbidity rate of up to 38%, traumatic carotid artery dissections and vertebral artery dissections are serious injuries in complex and severely injured patients [2,3,4]. The majority of these diseases remain initially asymptomatic, which might lead to a missed diagnosis [3,4] and avoidable delays in therapy such as anticoagulation, surgery, or endovascular treatment by thrombectomy or stenting. Traumatic cervical vessel dissections may result from rapid movement, both acceleration and deceleration, of the head in relation to the neck in any axis or blunt trauma.

In the daily routine, a contrast-enhanced multidetector computed tomography (CT) of the head, neck, thorax, abdomen, and pelvis according to the S3 guideline is used in severely and multiply injured patients and offers a demonstrable survival advantage [5], even when the mean injury severity score (ISS) was greater than that in patients without standardized imaging.

However, there are no specific guidelines on how to identify vascular injuries in the neck area as accurately as possible. In the current S3 guidline for the care of severely injured patients, for example, only vague recommendations for head-and-neck injuries are made [6]. Even highly experienced trauma surgeons, based on case history and clinical examination, miss ~50% of blunt injuries, stressing the need for standardized protocols and algorithms for imaging in trauma patients [7]. As a consequence, especially for head-and-neck injuries, more liberal screening criteria are discussed [8,9]. Technically, the diagnosis is challenging because characteristic imaging features can be difficult to detect because of the small size of intracranial and cervical arteries. Thus, multimodal imaging is often needed to safely confirm the diagnosis. Other than computed tomography angiogram (CT-A) and magnetic resonance angiography (MR-A) as the most widely used modalities for the detection of cervical artery pathologies, DSA (digital subtraction angiography) is considered the gold standard but is the most invasive option, requiring femoral arterial access and catheter placement in the cervical vessels, which is associated with a risk of hemorrhage and stroke. Ultrasound is the most inexpensive modality and can be performed at the bedside, but its diagnostic accuracy highly depends on the knowledge and experience of the user.

In single-bolus contrast-enhanced whole-body CT scans, traumatic lesions of the carotid and vertebral arteries often go unrecognized because of the low arterial contrast and contrast-enhanced parallel veins and soft tissues. Beam hardening artifacts at the skull base further worsen the detectability of such lesions, which mostly are discreet. If suspicion is raised, further diagnostic work-up is needed fast, causing additional radiation and contrast-agent dose in case of a CT-angiogram (CT-A) and time, cost, and delay in therapy of concomitant injuries. There is no widely accepted standard for addressing this dilemma in between overdiagnostics firsthand or missed diagnosis and stepped-up diagnostics later on. As a first step, the implementation of a non-dedicated angiogram revealed incidences of 6.5% for cervical artery dissection (CeAD), which were above reported values of 1.7% and 4.9% [8,10].

We thus decided that any patient qualified for a standard trauma CT scan, who has either been obviously severely injured or, according to trauma mechanism, is suspected to be severely injured, will receive a dedicated head-and-neck CT-A in between the non-contrast-enhanced brain scan and the delayed phase whole-body scan. As any injury to the head-and-neck region, especially vessel injuries such as carotid (CAD) or vertebral artery dissections (VAD), is of highest relevance for mortality, morbidity, and immediate therapeutic decisions, we accepted the additional radiation and contrast-agent trade-off based on an interdisciplinary consensus in our trauma center; this decision is in accordance with current recommendations of the Deutsche Röntgengesellschaft (German Radiology Society, [11]). After 18 months using the new single-bolus split-scan imaging approach, we here report the first results from our level I trauma center.

## 2. Materials and Methods

For this study, all patients admitted to the emergency room of our level I trauma center requiring a whole-body CT scan since November 2018 with an ISS ≥ 16 after complete diagnostic work-up were included. The decision for the CT scan is based upon the trauma mechanism and assumptions of its severity, e.g., based on estimated driving speed in car accidents, ejection from vehicle, falling from a great height (>3 m), burial trauma, death of co-driver, the observable injuries and circulation parameters in the emergency room, patient’s age, and assumed frailty, in accordance with national recommendations and guidelines as cited above. The decision is made by the leading trauma surgeon and radiologist on duty as a consensus. In November 2018, the whole-body CT scanning protocol was modified to a single-bolus split-scan approach including a dedicated contrast-enhanced head-and-neck CT-A triggered in the Aorta ascendens. The aim of this modification was to further optimize the detection rate of cervical artery dissections as we already had a non-dedicated head-and-neck angiogram as a mandatory component of our scanning protocol [4] with fixed-delay scanning.

The independent Ethics Committee at the Regensburg University confirmed that, for this specific scientific project, no dedicated ethics approval or commission’s opinion was necessary according to our applicable laws and guidelines. The consultation was filed under no. 20-1914-104.

### 2.1. CT Scan Protocol

All patients were examined using a 2 × 128-detector row dual-energy scanner (SOMATOM Definition Flash; Siemens Healthcare GmbH, Erlangen, Germany), fulfilling the requirements stated in the national S3 guideline “Polytrauma/Schwerverletzten—Behandlung” [6].

First, a non-contrast-enhanced scan of the neurocranium was acquired in 0.75 mm slices (360 mAs, 120 kV, pitch 0.55, increment 0.75 mm, and field of view 230 mm). The second scan is the contrast-enhanced head-and-neck CT-A, triggered in the A. ascendens. It reaches from the aortic arch covering the outlets of the supra-aortic branches up to the vertex. The volume and concentration of the contrast agent used (Accupaque 350, GE Healthcare Europe GmbH, Freiburg, Germany) were standardized to a total of 120 mL, with 12 injected for the head-and-neck CT-A. The contrast agent injection was followed by a flush with 20 mL of saline, both contrast agent and saline flush were injected with rates of 3.5 mL/s. ECG-gating is not routinely used in our protocol. The third scan was a venous phase delayed scan, covering the thorax, abdomen, pelvis, and upper half of the thigh; in select cases, the scan length was modified to cover the knees. It was scanned with a fixed delay of 30 s after the CT-A. The standard field of view was 500 mm, the slice thickness was 5.0 mm, the increment was 5.0 mm, the pitch was 0.60, kV and mAs were calculated and set automatically (CARE kV and CARE Dose; Siemens Healthcare GmbH) based on the scan topogram. A soft-tissue kernel with medium edge attenuation (B26f medium) was used for the calculation of axial, coronal, and sagittal views of the head and neck. Additional thick-slab axial maximal intensity projection (MIP) was rendered (10 mm) axial, sagittal, and coronal. Additional reconstructions were rendered using a B60f kernel for lung tissue (axial), a B60f sharp kernel for bones (axial and coronal, additional sagittal for spine), and a B30f kernel for soft tissue (axial, additional coronal for abdomen). Further reconstructions were calculated on the radiologists’ discretion depending on the findings or suspicions drawn from the standard datasets.

Image interpretation was performed using a standard three-monitor workstation using the Syngo and Syngo.via picture archiving and communication system (PACS; Siemens Healthcare GmbH, Erlangen, Germany). All imaging studies were read and validated by a board-certified radiologist. Additional magnetic resonance angiography (MR-A) and/or ultrasound were performed if the diagnosis of a cervical artery dissection remained unclear after the CT-A. Both MR-A and ultrasound were not mandatory parts of our diagnostic routine in the time period covered in this study.

### 2.2. Statistical Analysis

For statistical calculations, analysis, and plotting, GraphPad Prism Version 5.00a for Mac (GraphPad Software, Inc., La Jolla, CA, USA) and Microsoft Excel Version 16.38 (Microsoft Corporation, Redmond, WA, USA) were used. Arising from the observational character of this study, mainly, descriptive statistics was used. We did not calculate comparative statistics due to the small cohort size. Demographics and injury severity were compared against the cohort of all severely injured patients recorded in our database and/or the cohort of patients with CeAD before the most recent modification of our CT scan protocol, as reported before [4]. We calculated the confidence interval for proportions using a confidence level of 0.95. For prevalence or incidence proportions, in addition to the normal approximation interval, we calculated the Wilson score interval as it supports better results, especially for smaller samples and for edge proportions near 0 or 1 as in our case using R (V4.0.2, R Project, Vienna, Austria).

## 3. Results

Since introduction of our new CT scan protocol, 134 severely injured patients with an ISS ≥ 16 underwent the standardized whole-body computed tomography including a dedicated head-and-neck CT-A. Thereof, 7 patients were found with at least one cervical artery dissection (5.2%; 7/134). Six patients (85.7%; 6/7) had carotid artery dissections, with one patient having a CAD of both sides, and one patient having a CAD and contralateral VAD combined. Two patients (28.6%; 2/7) showed a VAD. ISS and mean age as well as gender distribution were comparable to past time frames (Table 1).

Another five patients underwent additional MRI including MR-A after CT with CT-A, which did not confirm a suspected cervical artery dissection. In 2 out of 7 cases with CT-A-diagnosed CeAD reported here, MRI confirmed the diagnosis. In 5 cases, CT-A diagnosis required no further imaging work-up. In all, 3 of 7 patients had an ultrasound of the extracranial vessels but none was able to identify the dissection; two of them had an MR-A-confirmed diagnosis of CeAD.

On admission, none of the patients showed any neurologic symptoms attributable to a CeAD. Five patients were put on anticoagulation (71.4%), two patients were not eligible for anticoagulation due to either intracerebral hemorrhages or a severe distraction-flexion-type spinal fracture C4–7. Two patients developed serious neurologic symptoms (aphasia, hemiparesis) due to carotid artery dissections, yielding a morbidity of 28.6%. One patient died, yielding a mortality of 14.3%.

**Figure 1:**
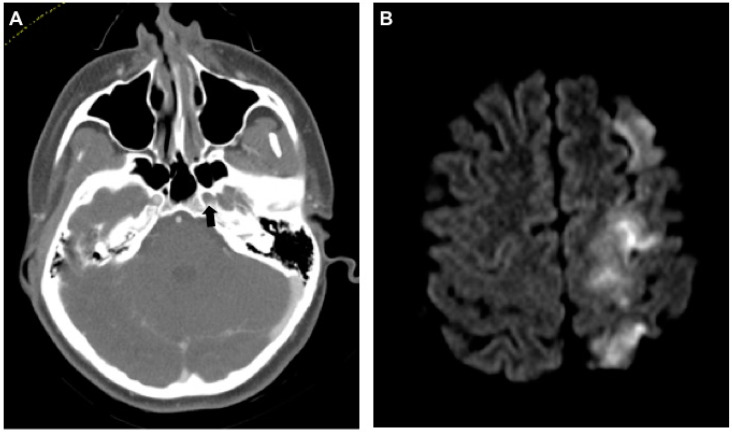
Dissection of the left internal carotid artery (ICA) with severe neurologic sequelae. (**A**) Axial computed tomography angiogram (CT-A) of the skull base shows no contrast agent in the ICA within the foramen carotideum on the left (black arrow). (**B**) Same-day magnetic resonance imaging (MRI) (diffusion weighted imaging (DWI), b800) shows ipsilateral infarction of the anterior and middle cerebral artery territories.

The trauma mechanism was a fall from large height in one case (14.3%) and traffic accidents in all other cases (4 car accidents, 1 motor-scooter, 1 e-bike; 85.7%). Concomitant spinal fractures were found in 4 cases (57.1%). One patient had a complex midface fracture reaching into the skull, one patient had no fracture of the spine or thorax (Figure 1). Three patients had fractures of upper third ribs (42.9%).

## 4. Discussion

In severely injured patients, rates of cervical artery dissections are reported to be as low as 0.9% based on ultrasound [12] and said to occur in around 1% of patients with blunt trauma [13]. Recently, we found a six-fold higher rate of 6.5% in our level I trauma center population by making a head-and-neck angiogram a mandatory component in the CT scan protocol for severely injured patients admitted to the emergency room [4]. This led to the idea, that further optimization of our scan protocol could yield even higher rates of cervical artery dissections and lower rates of non-diagnosed cases of cervical artery dissection, as a relevant proportion never shows any neurologic symptoms but is at risk for severe sequelae firsthand. We thus switched our scan protocol to a single-bolus split-scan scheme, which enabled integrating a dedicated head-and-neck CT-A into our diagnostic routine. Moreover, 18 months after implementing the new protocol into daily routine of care, we now can report a rate of 5.2%, which is lower than the 6.5% reported before, but higher than the 4.5% from the time we did not use a scanning protocol optimized for the detection of cervical artery dissections and still five-fold the value estimated overall for blunt traumata [13]. At the same time, we had 7 cases with an additional MRI/MR-A; five in patients with suspected dissection based on the CT-A, which all were negative; two in patients with a certain diagnosis of cervical artery dissection after CT-A with the primary intent of revealing the cerebral and cerebellar ischemic impacts.

Different conclusions can be drawn from the observations made: first, the true rate of cervical artery dissections in severely injured patients is likely to be in between our reported values of 6.5 and 5.2% in our trauma center and seems to vary rather widely within different populations as reported from different trauma centers worldwide; the Wilson score interval, which is a variant of the normal approximation 95% confidence interval more suitable for small sample sizes and edge proportions near 0 as in our case, yields a 95% CI range between 2.6% and 10.4% for CeAD in this cohort.

**Figure 2:**
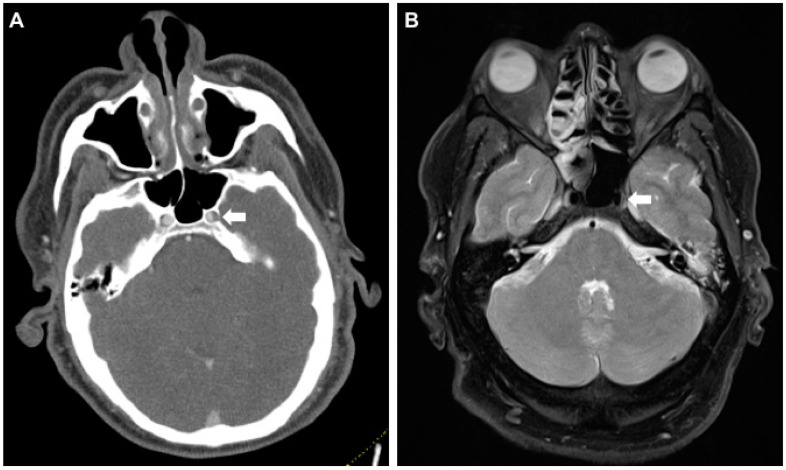
False-positive CT-A raising suspicion for an intramural hematoma of the left internal carotid artery (ICA) in its petrosal segment. (**A**) Axial CT-A of the skull base shows caliber asymmetry of the left ICA in the foramen carotideum (white arrow). (**B**) MR (proton-density fat saturated) rules out a wall hematoma. Asymmetry is presumably caused by the carotid plexus of nerves and sympathetics from the superior cervical ganglion (white arrow). MR, magnetic resonance.

Second, dedicated CT-A did not lead to more dissections revealed in our cohort but did elicit uncertain or suspicious cases requiring additional imaging (Figure 2).

Third, the most powerful predictor for cervical artery dissections seems to be any kind of high kinetic trauma mechanism, especially traffic accidents including cars, motorcycles/-scooters, bikes, and e-bikes, which is also true for aortic arch injuries due to a “whiplash”-type motion of fast deceleration and re-acceleration [14,15]. Some fractures should raise high suspicion toward cervical artery injuries, such as fractures of the cervical spine, skull base and/or complex midface fractures, or upper third rib (costae 1–4) fractures. Nonetheless, devastating dissections without an accompanying fracture are possible (Figure 1).

Fourth, large multicenter or registry-based studies are required to accurately assess cervical artery dissection risk factors, outcomes, and characteristics, as it will remain a low-incidence injury and rates between 1% [13] and 6.5% [4] are reported repeatedly.

Fifth, multimodal work-up of suspicious cases is highly relevant as with a dedicated head-and-neck CT-A a tendency toward false positives can be seen. With ultrasound, which often is used as the first add-on diagnostic due to fast availability and low cost, false negatives are very likely [12], being unable to safely rule out any CeAD and, as in our cases reported here, not being able to confirm CeAD either. Influence of dedicated training of ultrasound examiners or compulsory use of state-of-the-art techniques such as b-flow or contrast-enhanced ultrasound should be elaborated.

Despite the low incidence, the diagnosis of any artery dissection has to be made fast due to the severe consequences from any therapeutic delay, especially in cervical vessels as central nervous ischemia or embolization can lead to devastating and irreversible neurologic sequelae. In a population aged 45 and younger, 20% of ischemic strokes are due to traumatic and non-traumatic CeAD [12]. Moreover, blunt carotid injury has been associated with a higher stroke rate in general (up to 60%) and mortality rate (19–43%) [16]. In cases of cervical artery dissections, besides open surgery, recanalization via flow diverting or covered stenting of the carotid artery can be an option [17,18], with successful cases reported even for the vertebral arteries [19]. Anticoagulation should be established to prevent thrombus formation and embolization. However, in patients with ISS 16 and above, the majority show fractures, bleedings, or abdominal organ lacerations, which might stand in contradiction to systemic anticoagulation [20]. In these cases, an individual interdisciplinary treatment regime needs to be defined balancing possible benefits and risks. Crönlein et al. mentioned accordingly that there is no specific guideline for diagnosis and treatment established when it comes to severely injured patients, which is applicable to our patient collective [21]. Current recommendations from the German society for radiology suggest implementing differentiated scanning protocols, adjusted for dose and/or time; both include a cervical CT-A [11] despite the lack of larger trials or investigations addressing the topic of imaging optimization in the care of severely injured patients.

Injuries of the carotid or vertebral artery are reportedly associated with both high mortality rates and neurological morbidity rates, as again seen in the cohort reported here. In severely injured patients, diagnoses and decisions only based on history and clinical examination are prone to error with missed injuries in up to 47%. As none of the patients with CeAD in this population showed symptoms hinting specifically at a cervical vessel injury on admission, these diagnoses most likely would have gone unrecognized until the development of neurologic symptoms. Interestingly, a significant number of (66–73%) patients with blunt carotid injury may be asymptomatic upon initial presentation, developing delayed neurologic symptoms anywhere from 1 h to 7 days after injury. The onset of ischemic events can range from a few minutes to 31 days after injury, with the majority (82%) occurring within the first 7 days [9,22].

This delay in therapy needs to be avoided under all circumstances as secondary worsening or complications such as cerebral embolization could be prevented. Occlusive CeAD, multiple CeAD, and vertebral artery dissections result in an increased risk for delayed stroke, as reported by Lichy et al. [23].

Comparing the rates reported here with larger studies, our values are above those of others, possibly resembling missed injuries due to non-specific imaging protocols. Nevertheless, our values are lower than those reported from clinically preselected cohorts and our own past cohort. As a comparison, Lee et al. report an average annual incidence rate for CeAD of any cause of 2.6 per 100,000 population (95% CI, 1.86 to 3.33) [24]. Major trauma has an estimated incidence rate of 22.5 per 100,000 population in Germany [25], which leads us to an estimated incidence rate of 1.2 per 100,000 population (95% CI, 0.33 to 2.02) for CeAD in severely injured patients assuming our latest rate of 5.2%.

### Limitations

As cervical artery dissections are highly relevant for the severely injured individual admitted to any emergency room, we wanted to report here the first results from our modifications of our diagnostic routine with the intent to nurture further discussions on the best diagnosis and treatment regimes in these uncommon but regularly to be seen cases. Thus, some relevant limitations are inherent to our study design.

First, as a single-center study reporting from an 18 month period, only few cases could be found and included. This needs to be addressed by further multicenter or registry-based examinations. As a direct consequence, we decided to report descriptive statistics and to not include comparative statistics, as this could tempt readers to draw conclusions that are not backed by sufficient data as this is a preliminary report. Second, in our level I trauma center, severely injured patients are admitted at a rate above the nationwide average in both severity and frequency, due to geographic and strategic reasons within the local emergency medicine services. Considering this, other hospitals will likely find lower or even higher incidences of CeAD in their population. Third, the true incidence of cervical artery dissections still remains unknown; no case showed neurologic symptoms on admission, some dissections remain clinically inapparent (3 out of 7 in this study), and a definite multimodality diagnostic work-up for suspected cases or even all severely injured patients is not yet established. In consequence, further studies should address the question of true incidence of CeAD by multimodality work-up including MR-A. However, capped capacities for additional MR-A work-up especially in smaller hospitals is another limitation that needs to be considered in setting up a diagnostic pathway for severely injured individuals.

The sensitivity (64–100%), specificity (67–100%), positive predictive value (65–100%), and negative predictive value (70–100%) of CT-A for the detection of CeAD compared with those of DSA vary widely with later studies indicating values close to those of DSA due to essential technical improvements made in the last few years [26,27]. MR-A has a sensitivity of 50–100%, specificity of 29–100%, positive predictive value of 43–100%, and negative predictive value of 89% compared to those of DSA [28]; especially for the smaller vertebral arteries, MRI yields diagnostic accuracy below that of CT-A because of the limited spatial resolution in many MRI/MR-A protocols. With modern scanners, the highest spatial resolution of MRI is at the same level as that of CT. Limited availability and long duration of the image acquisition in combination with physical restrictions (metal implants, narrow gantry) still makes MRI/MR-A not an option for first-line diagnostics in cases of suspected traumatic CeAD, as it is recommended as an initial test in non-trauma cases by the American Stroke Association (ASA) and American College of Radiology (ACR) [29].

One key finding of the results reported here is the need for a diagnostic routine in severely injured patients that is able to reliably detect cervical artery injuries, as none of the patients covered in the recent 18 month period showed symptoms on admission. Whenever possible, we put patients with CeAD on anticoagulation based on a case-by-case interdisciplinary consensus. Whether the low morbidity and mortality in comparison to cohorts reported before can be attributed to our imaging pathway cannot be answered from this study reasonably due to the limitations discussed before. As a first measure, any suspected case of cervical artery dissection should be ruled out by MRI/MR-A and/or qualified ultrasound within a defined and short timeframe (e.g., 2 h) based on the high mortality and morbidity risk in un- or delayed diagnosed cervical artery dissections.

## 5. Conclusions

Modification of the trauma imaging routine to include a dedicated head-and-neck CT-A realized as a single-bolus split-scan protocol showed an incidence of 5.2% for cervical artery dissections after the first 18 months under operation, which is slightly below values reported before, as are both mortality (14.3%) and morbidity (28.6%) in this cohort. We repeatedly encountered false-positive findings requiring further diagnostic work-up with MR-A. Ultrasound did not help substantially to make or rule out the diagnosis of CeAD in our setting. Further studies will need to address the value of multimodality diagnostics and the role of state-of-the-art ultrasound techniques for extra- and intracranial vessel diagnostics. As none of the detected cases of cervical artery dissections showed neurologic symptoms on admission, a reliable, fast, and multimodal imaging pathway for the detection of cervical artery dissections in severely injured patients is of utmost importance in trauma centers of any size to avoid any therapeutic delay.

## Figures and Tables

**Table 1:** Cohort characteristics depending on the diagnostic approach for the detection of cervical artery dissections (CeAD).

	CeAD W/O Dedicated Scan Protocol	CeAD W/Mandatory Angiogram	CeAD W/Dedicated CT-A
*N* (*n*)	53 of 1178	15 of 230	7 of 134
Rate of CeAD (%)	4.5	6.5	5.2
CI95	0.033; 0.057	0.033; 0.097	0.015; 0.090
Wilson score interval (CI95)	0.035; 0.058	0.040; 0.110	0.026; 0.104
ISS (mean)	39	35	33
Age (mean)	48	51	48
Sex (m/f)	36/17	10/5	5/2

CI95 = 95% confidence interval; ISS = Injury Severity Score; CT-A = computed tomography angiogram.
